# Measuring the Effects of Cannabis on Anxiety and Depression Among Cancer Patients

**DOI:** 10.1002/cam4.71342

**Published:** 2025-10-29

**Authors:** Apoorva C. Reddy, John M. Hampton, Susan J. Park, Faith Dickerson, Janvi Shah, Betty Chewning, Natalie Schmitz, Amy Trentham‐Dietz

**Affiliations:** ^1^ Department of Surgery, School of Medicine and Public Health University of Wisconsin‐Madison Wisconsin USA; ^2^ Department of Population Health Sciences and Carbone Cancer Center, School of Medicine and Public Health University of Wisconsin‐Madison Wisconsin USA; ^3^ Minnesota Office of Cannabis Management Analytics Division Minnesota USA; ^4^ School of Pharmacy University of Wisconsin‐Madison Wisconsin USA

**Keywords:** anxiety, cancer, cannabis, depression, longitudinal studies, palliative care

## Abstract

**Introduction:**

Cancer patients are increasingly turning to cannabis products to modulate physical and psychological symptoms despite limited evidence supporting their efficacy. We aimed to explore cancer patients' self‐reported anxiety and depression symptoms in response to cannabis use.

**Methods:**

This longitudinal study examined how patient‐reported anxiety and depression symptoms varied according to the dose, ratio of tetrahydrocannabinol (THC) to cannabidiol (CBD), and route of administration of cannabis products among cancer patients. Change in self‐reported anxiety and depression symptoms was evaluated in 1962 cancer patients after 30 days of enrollment in the Minnesota Medical Cannabis Program.

**Results:**

Anxiety scores improved more in patients taking higher doses of CBD (> 14.3 mg/day) compared to those taking lower doses (< 4.6 mg/day) and among patients using enteral cannabis products. Depression scores also improved more for patients taking enteral products.

**Discussion:**

Anxiety scores varied according to the dose of cannabis, the ratio of THC to CBD, and the route of administration of cannabis products. In contrast, depression scores only varied according to the route of administration.

**Conclusions:**

This study of cancer patients in Minnesota suggests that patterns of cannabis use that include relatively higher doses of CBD taken enterally may improve the quality of life of cancer survivors who report anxiety and depression. This study constructs a foundation for future research to improve the tailoring of cannabis‐related educational materials to patients' needs and inform the training of healthcare professionals on how to recommend cannabis products for cancer survivors.

## 1 Background

Cancer patients were among the first to receive access to medical cannabis products for symptom management due to the high symptom burden resulting from cancer and its treatment [1, 2, 3]. Since 1996, an increasing number of cancer patients have been using cannabis for symptom management despite limited evidence of its effectiveness and unknown risks of adverse effects [4, 5, 6]. Clinical trials have demonstrated that cannabis can provide relief from physical symptoms in patients with cancer; this paved the way for the United States (US) Food and Drug Administration to approve cannabis product use for chemotherapy‐induced nausea and vomiting [7, 8]. However, cancer patients commonly report using cannabis to manage psychological symptoms like anxiety and depression [9]. One national surveillance study found that anxiety and depression were common complaints among cancer survivors, at 46% and 20%, respectively [10]. With over 2 million cases of cancer estimated in the US for 2025 and up to one‐third of cancer patients reporting cannabis use in the past month, research is urgently needed to clarify the impact of cannabis on psychological symptoms [4, 11].

Clinical trial‐based evidence is insufficient to support the use of cannabis for anxiety and depression; however, tetrahydrocannabinol (THC) and cannabidiol (CBD) might impact mood‐related symptoms [9]. These compounds, derived from the cannabis plant, interact with receptors in the endocannabinoid system, which plays a critical role in regulating mood [12, 13]. Both THC and CBD interact with CB1 and CB2 receptors, which are part of the endocannabinoid system [14, 15]. CB1 and CB2 both have receptors in the central nervous system (CNS) contributing to psychological and behavioral effects. The majority of the CB1 receptors are located in the CNS, while CB2 receptors mostly contribute to immune‐modulating activity and are located on immune cells, hemopoietic cells and the spleen [16].

Cannabinoids affect the neurotransmissions of gamma‐aminobutyric acid (GABA) and glutamate and enhance the activation of serotonin pathways through serotonin (5HT) receptors. THC is recognized for elevating dopamine and glutamate levels and reducing GABA in the prefrontal cortex [17]. CBD is thought to mitigate the psychoactive effects of THC by acting as a noncompetitive antagonist at the CB1 receptors. Additionally, CBD is an agonist at the serotonergic receptors and an antagonist at CB2 receptors [9]. Preliminary research suggests that CBD's modulation of various pathways (e.g., endocannabinoid, serotonergic, and glutamatergic systems) may contribute to its potential to reduce anxiety and depressive symptoms [12, 18, 19]. Like CBD, THC interacts with serotonergic systems, with low doses of THC stimulating the release of serotonin, which may help alleviate symptoms of depression [20].

While CBD is known for its anxiolytic properties, THC exhibits a more complex relationship with anxiety, with preclinical studies indicating a biphasic, dose‐dependent effect [18, 21]. While the therapeutic potential of cannabinoids is promising, particularly for addressing anxiety and depressive symptoms in cancer patients, strong evidence is lacking [9].

Cannabinoid dose is not the only factor affecting symptom response. Anxiety and depression symptom responses are thought to also vary according to the ratio of THC to CBD and the route of administration of cannabis products [9]. The ratio of THC to CBD may be important because CBD and THC may differentially interact with receptors, thereby producing different psychiatric effects. For example, it is thought that CBD is a negative allosteric modulator of CB1 receptors, for which THC is an agonist [22, 23]. CB1 receptor antagonists can be anxiogenic and induce a depressive phenotype [24]. Additionally, the route of administration may affect the association between cannabinoids and symptoms due to variations in onset times and durations of effects. For example, the impact of enteral products tends to be the most delayed relative to other routes of administration because they must be digested before cannabinoids can enter the bloodstream [25].

The lack of knowledge surrounding cannabinoid pharmacodynamics impedes both patients and providers in determining the best cannabis products for managing symptoms. To address this knowledge gap, the objective of this longitudinal study was to quantify the change in reported symptoms of anxiety and depression in patients with cancer entering a statewide medical cannabis program.

## 2 Methods

### 2.1 Study Setting

The setting for this study was the Minnesota Medical Cannabis Program (MMCP). MMCP provides a treatment option for patients with certain qualifying conditions to access cannabis [26]. Healthcare practitioners certify whether a patient has an MMCP‐approved qualifying condition and refer patients to the program. Once enrolled in the program, patients can visit the MMCP dispensary locations. During their visit to the MMCP dispensaries, patients consult with the dispensary pharmacist to determine the appropriate cannabis formulation, route of administration, and dose regimen based on their qualifying condition, symptoms, previous use of cannabis, goals for therapy, and other medications or products used. Patients complete a Patient Self‐Evaluation survey before every purchase through an online patient registry at the registered medical cannabis dispensary. One goal of the Patient Self‐Evaluation is to provide documentation of any achieved stabilization of the patients' symptom scores by the 30‐day mark following enrollment in the MMCP [27, 28].

### 2.2 Data Collection

Data for this study came from three sources: patient applications to enroll in the MMCP, Patient Self‐Evaluation surveys, and dispensary transactions. Patients provided information on their demographics, including age, race, ethnicity, sex, and whether they qualified to pay a reduced fee to enroll in the MMCP. Patients qualified for the reduced fee at some point during the study period in the MMCP if they were enrolled in any of the following: Civilian Health and Medical Program of the United States Department of Veterans Affairs (CHAMPVA), Veterans Affairs medical assistance for disability, Railroad disability, Social Security Disability (SSDI), Supplemental Security Income (SSI), enrollment in medical assistance (MA) or MinnesotaCare or Medicaid, Indian Health Services enrollment, and Veterans Affairs disability. The reduced fee was $50, and the standard cost was $200.

On the Patient Self‐Evaluation form, patients provided weight, height, and rated their symptoms of anxiety and depression on an ordinal scale of 0–10, where 0 indicated the symptom had not been present, and 10 indicated the symptom was as bad as you can imagine it could be in the preceding 24 h [28, 29]. This scale was based on the Edmonton Symptom Assessment System (ESAS) symptom battery, which was validated for use with cancer patient populations [30].

The cannabis purchase transaction data included the amount (mg) of THC and CBD contained in each purchased product along with the quantity (units) purchased and the estimated duration (days) the pharmacist anticipated the product would last before a refill was needed based on the instructions for use. The frequency of refills varied, with some patients visiting MMCP dispensaries for refills or new products more frequently than others. The products sold at the dispensaries were purchased through two manufacturers, RISE (Leafline Labs) and Green Goods (rebranded from Minnesota Medical Solutions and Vireo Health). Each manufacturer leads its own cultivation, production, and distribution of medical cannabis in Minnesota.

Products for inhalation, enteral, oromucosal, and topical routes of administration were included. Inhalation products include combusted products that are inhaled through the mouth and absorbed via the lungs (e.g., dried flower and vapes). Enteral products are those that are swallowed and absorbed via the gastrointestinal tract (e.g., tablets, capsules, gummies, oral suspensions, and oral solutions). Oromucosal products include products that are held in the mouth or under the tongue and absorbed via the oromucosal lining (e.g., sublingual sprays, tinctures, lozenges, and oral spray). Topical products include products applied to the surface of the body and skin (e.g., creams, lotions, balms).

### 2.3 Statistical Analysis

Analysis of study data was informed by the Gelberg–Andersen Behavioral Model for Vulnerable Populations to explore the relationship between cannabis product characteristics and change in self‐reported symptom severity after 30 days of enrollment in the MMCP [31]. Appendix A describes the application of the Gelberg–Andersen Behavioral Model for Vulnerable Populations and the definitions of each variable included in the analysis. A patient's choice of cannabis regimen and their symptom response to that regimen frequently varied after initiating use but was expected to stabilize within 30 days of enrollment [27]. Cannabis purchasing transaction data were used as a proxy for the actual use of cannabis products. Product characteristics of interest included average daily cannabinoid dose, THC:CBD ratio, and route of administration. If a patient purchased multiple products at once, each product's cannabinoid quantity was divided by the day's supply and then summed to calculate the average dose per day (mg). Values for product characteristics in the analysis were based on the first visit after 30 days since characteristics tended to vary widely within the first month in the MMCP. Raw cannabis (e.g., dried flower products), which became available in 2022, was excluded from the analysis because the quantification process for the THC and CBD contents in flower had yet to be set. If a patient purchased raw cannabis, only their purchases prior to that transaction were eligible for inclusion in the analysis.

Symptoms of interest included self‐reported anxiety and depression. Since the primary outcome of interest was the change in symptom severity after enrollment in the MMCP, the difference was calculated in the symptom severity scores between enrollment and the average of symptom scores after 30 days. At least two post–30 day visits were required since cannabis characteristics were examined based on the first transaction after 30 days, and symptom scores were used after that transaction, that is, those that could reflect the effects of the associated cannabis use. For patients with at least three post–30 day Patient Self‐Evaluation surveys, symptom scores were averaged since the number of visits varied by patient (excluding the first survey after 30 days, since cannabis characteristics were based on that visit). If a patient had a gap of more than 120 days between visits, only their symptom scores before that gap were included in the analysis. The largest day supply from an MMCP cannabis purchase transaction was 120 days. Thus, the 120 day limit prevented the misattribution of changes in symptom scores to unrelated cannabis products or use characteristics.

Descriptive statistics were used to describe demographic data of patients, patterns of cannabis use, and reports of patient symptoms. The frequency of average daily doses, THC: CBD ratios, and routes of administration were enumerated. Logistic regression models were used to estimate odds ratios (OR) and 95% confidence intervals (CI) of a clinically meaningful (≥ 30%) symptom score improvement (on a scale of 0–10) according to quantities of THC, quantities of CBD, and ratios of THC:CBD achieved. A 30% improvement was chosen as the conservative estimate of the clinically significant improvement, based on previous studies showing that ≥ + 1 on the ESAS was an optimal Minimal Clinically Important Difference (MCID) cut‐off for anxiety and depression [30, 32]. Multivariate linear regression models were used to evaluate the association of THC and CBD use patterns with the reported symptom scores for anxiety and depression. Regression models were adjusted for age, sex, race, ethnicity, body mass index (BMI, kg/m^2^), baseline symptom score, and MMCP enrollment fee. Values above the 95th percentile for BMI were set at the 95^th^ percentile value for regression modeling. Graphs were created from separate analyses for the predicted probability of achieving a 30% or greater improvement in anxiety and in depression for average dose per day (mg) for THC and CBD using restricted cubic spline with knots at 5, 27.5, 50, 72.5, and 95 percentiles. Similar graphs were created for the predicted improvement in anxiety and in depression. All analyses were conducted using the statistical software Statistical Analysis System (SAS Institute Inc.).

## 3 Results

### 3.1 Study Population for Analysis

**FIGURE 1:**
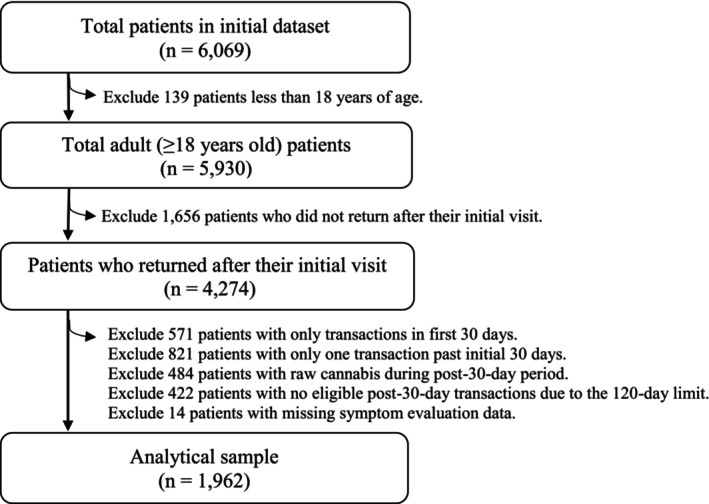
Flowchart of sample for analysis.

Patients with cancer were eligible for the MMCP if a healthcare practitioner referred them and reported that they were experiencing severe or chronic pain, nausea or severe vomiting, or cachexia or severe wasting. The experience of anxiety or depression was not a certifiable symptom or condition for patients to enroll in the MMCP. The study population included adult patients with cancer (*N* = 6069) in the MMCP between July 1, 2015, and June 9, 2023 (Figure 1). As described above, patients were included in the analytical sample if they were adults and enrolled in the MMCP for at least 30 days (*N* = 4274). After additional exclusions based on requirements for the statistical analysis, the analytic sample included 1962 individuals.

### 3.2 Patient and Cannabis Use Characteristics

The average age of patients in this study was 57.4 years (standard deviation, SD: 13.9 years). The average BMI was 26.9 kg/m^2^ (SD: 6.3 kg/m^2^) at MMCP enrollment. Most patients in the sample were 50 years of age or older (*n* = 1473, 76%), with males comprising 51% and females 49% of the sample (Table 1). Most patients were White (89.1%) and non‐Hispanic (95.3%), and 40% of the sample paid the reduced enrollment fee for the MMCP. Only 6% of the sample reported having metastatic cancer. Patients visited the MMCP a median of five times with an average enrollment time of 10.5 months.

**TABLE 1:** Sample characteristics of cancer patients in the Minnesota Medical Cannabis Program (*N* = 1962).

Age (years)	*N*	%
18–39	239	12.2
40–49	250	12.7
50–59	504	25.7
60–69	603	30.7
70–79	286	14.6
≥ 80	80	4.1
Sex		
Female	961	49.0
Male	1001	51.0
Race		
White	1748	89.1
Black	55	2.8
Asian	27	1.4
Native American	40	2.0
Other/Multiple	33	1.7
No answer/unknown	59	3.0
Ethnicity		
Not Hispanic	1870	95.3
Hispanic	38	1.9
No answer/unknown	54	2.8
Metastatic cancer		
Confirmed yes	117	6
No/not reported	1845	94
MMCP enrollment fee		
Reduced price ($50)	791	40.3
Full price ($200)	1171	59.7

Enteral products were the most used route of administration, with 75% of cancer patients using at least one enteral product. The most common combination of routes of administration used during MMCP enrollment was enteral and inhalation products (19%, Table 2). However, the exclusive use of enteral products remained greater than any combination, at 25%. Patients took, on average, 32 mg of THC per day (SD: 71 mg) with a median dose of 17 mg per day, and 14 mg of CBD per day (SD: 44 mg), with a median dose of 3 mg per day. On average, enteral products had the lowest amounts of THC compared to products in any of the other categories of routes of administration. The median THC to CBD ratio of cannabis products used per day by MMCP patients was 7.21.

**TABLE 2:** Predicted improvement in self‐reported anxiety and depression scores according to cannabis route of administration.

Route of administration	*N*	%	Anxiety results			Depression results		
			predicted improvement (Score range: 0–10) *	95% CI	*p*	predicted improvement (Score range: 0–10) *	95% CI	*p*
Enteral								
None	487	24.8	1.16	0.92–1.40	ref.	1.10	0.87–1.33	ref.
Any	1475	75.2	1.53	1.38–1.68	0.004	1.36	1.22–1.51	0.04
Inhalation								
None	918	46.8	1.27	1.06–1.48	ref.	1.25	1.05–1.45	ref.
Any	1044	53.2	1.42	1.24–1.60	0.24	1.21	1.03–1.39	0.73
Oromucosal								
None	1368	69.7	1.33	1.16–1.50	ref.	1.20	1.03–1.36	ref.
Any	594	30.3	1.36	1.15–1.57	0.84	1.27	1.06–1.47	0.52
Topical								
None	1601	81.6	1.33	1.20–1.47	ref.	1.12	0.98–1.25	ref.
Any	361	18.4	1.36	1.10–1.61	0.86	1.35	1.10–1.59	0.08
Route Combinations								
Enteral only	483	24.6	1.40	1.19–1.62	ref.	1.18	0.97–1.39	ref.
Enteral + Inhalation	378	19.3	1.58	1.35–1.82	0.28	1.24	1.01–1.47	0.71
Inhalation only	289	14.7	1.24	0.96–1.52	0.39	0.94	0.66–1.21	0.18
Enteral + Oromucosal	197	10.0	1.60	1.27–1.92	0.31	1.38	1.06–1.70	0.29
Enteral + Inhalation + Oromucosal	119	6.1	1.60	1.17–2.02	0.42	1.19	0.78–1.60	0.97
Enteral + Inhalation + Topical	87	4.4	1.28	0.80–1.77	0.66	1.19	0.72–1.67	0.95
Enteral + Topical	83	4.2	1.79	1.29–2.29	0.15	1.80	1.31–2.29	0.02
Oromucosal only	76	3.9	0.93	0.41–1.45	0.10	0.78	0.27–1.29	0.15
Other combinations	250	12.7	1.36	1.08–1.65	0.83	1.35	1.07–1.63	0.33

*Note:* *Adjusted for age, sex, race, Hispanic ethnicity, BMI, baseline symptom score, MMCP enrollment fee, and THC: CBD ratio.

Abbreviations: CI, confidence interval; ref, reference group.

### 3.3 Anxiety Symptoms

**FIGURE 2:**
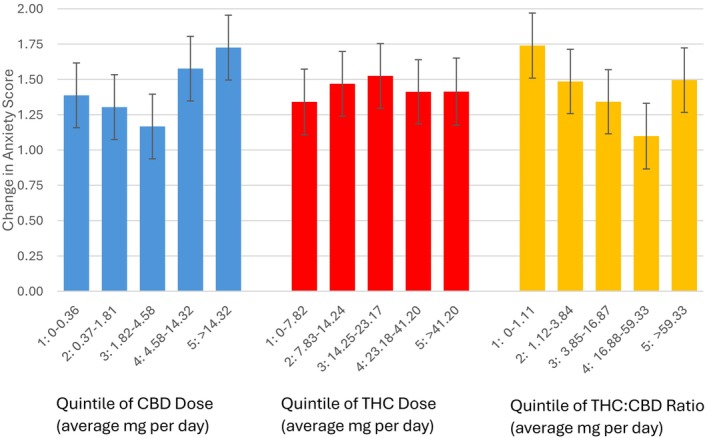
Quintile of CBD dose, THC dose, and THC to CBD ratio in relation to change in self‐reported anxiety score. The change in anxiety score refers to the reduction in score on a scale of 1–10.

The average percentage improvement in anxiety (i.e., reduction in patient anxiety scores) was 24.8%. The improvements in self‐reported anxiety scores were positively associated with the CBD dose but not the THC dose of cannabis products. For each 5 mg increase in the daily average CBD intake, the patient's anxiety score improved by 0.05 points (95% confidence interval 0.01–0.08, *p* = 0.01). Compared to patients in quintile group 5 with the highest dose of CBD, patients with lower CBD doses were less likely to report at least 30% improvement in anxiety scores (quintile 2 *p*: 0.01, CI: 0.51–0.92; quintile 3 *p*: 0.05, CI: 0.49–0.88; Appendix B, Table A1). In other words, patients in the fifth and highest CBD quintile had a predicted improvement in anxiety scores of 1.73 out of 10 points, which was significantly greater than score improvements in patients in the lowest three quintiles of average daily CBD dose (Figure 2).

Changes in anxiety scores appeared similar across quintile groups of THC dose (Appendix B, Table A2). Similarly, THC: CBD ratio was not strongly associated with a consistent trend in reported anxiety (Figure 2). Appendix B, Figure A3 shows graphs of the predicted probability of achieving a 30% or greater improvement in anxiety for average dose per day (mg) for THC and CBD using restricted cubic spline with knots at 5, 27.5, 50, 72.5, and 95 percentiles. Appendix B, Figure A4 shows graphs of the predicted improvement in anxiety.

Enteral was the only route of administration associated with improvement in anxiety. Patients who took any enteral products reported a 1.53 reduction in anxiety scores on a 10‐point scale compared to a 1.16 reduction for those who did not (*p* = 0.004) (Table 2).

### 3.4 Depressive Symptoms

**FIGURE A2:**
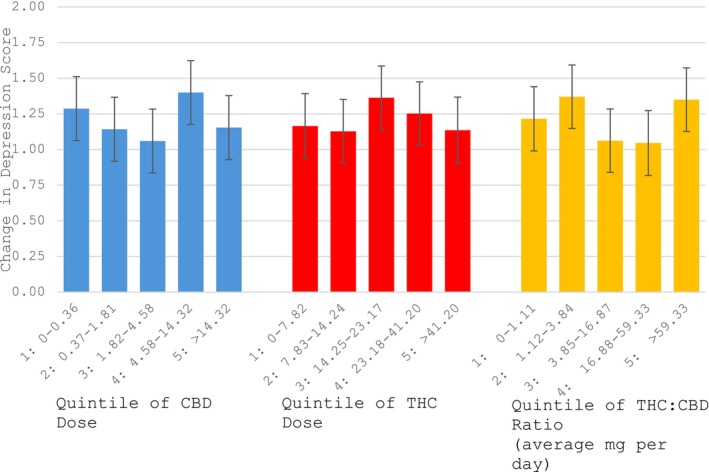
Quintile of CBD dose, THC dose, and THC to CBD Ratio in Relation to Change in Self‐Reported Depression Score. The change in depression score refers to the reduction in score on a scale of 1–10.

**FIGURE A3:**
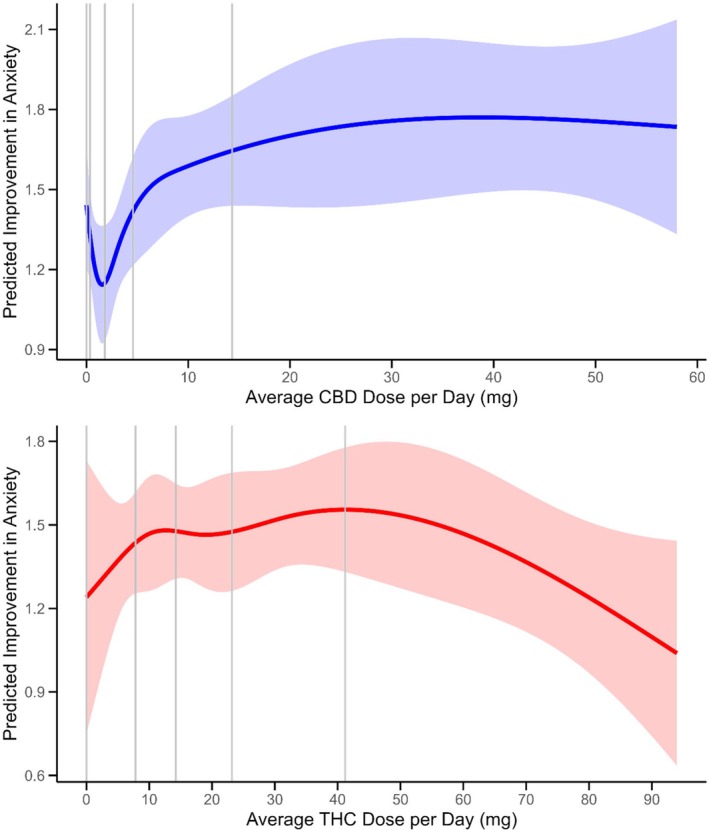
Predicted Improvement in Disturbed Sleep for Average Dose per Day (mg) for THC and CBD. Predicted values using restricted cubic spline with knots at 5, 27.5, 50, 72.5, and 95 percentiles are shown with 95% confidence limits. Vertical gray lines mark quintile cutpoints for average dose.

**FIGURE A4:**
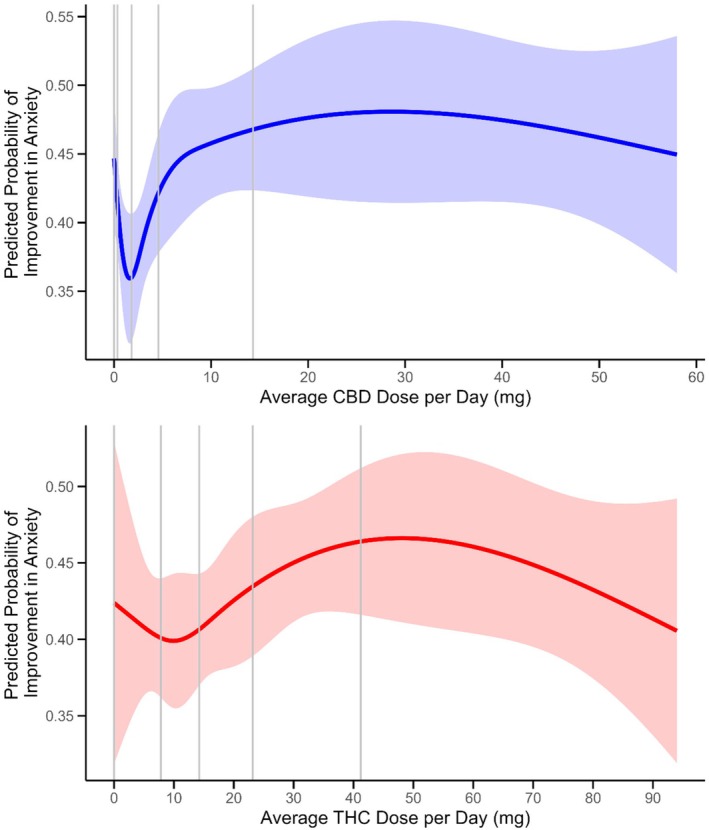
Predicted Probability of Achieving a 30% or Greater Improvement in Disturbed Sleep for Average Dose per Day (mg) for THC and CBD. Predicted probability using restricted cubic spline with knots at 5, 27.5, 50, 72.5, and 95 percentiles are shown with 95% confidence limits. Vertical gray lines mark quintile cutpoints for average dose.

**FIGURE A5:**
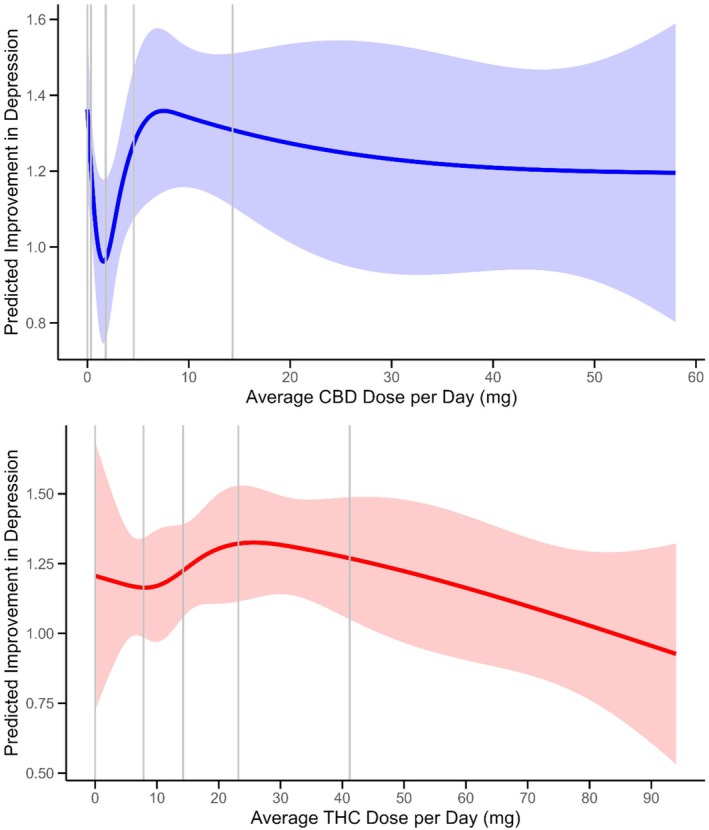
Predicted Improvement in Disturbed Sleep for Average Dose per Day (mg) for THC and CBD. Predicted values using restricted cubic spline with knots at 5, 27.5, 50, 72.5, and 95 percentiles are shown with 95% confidence limits. Vertical gray lines mark quintile cutpoints for average dose.

**FIGURE A6:**
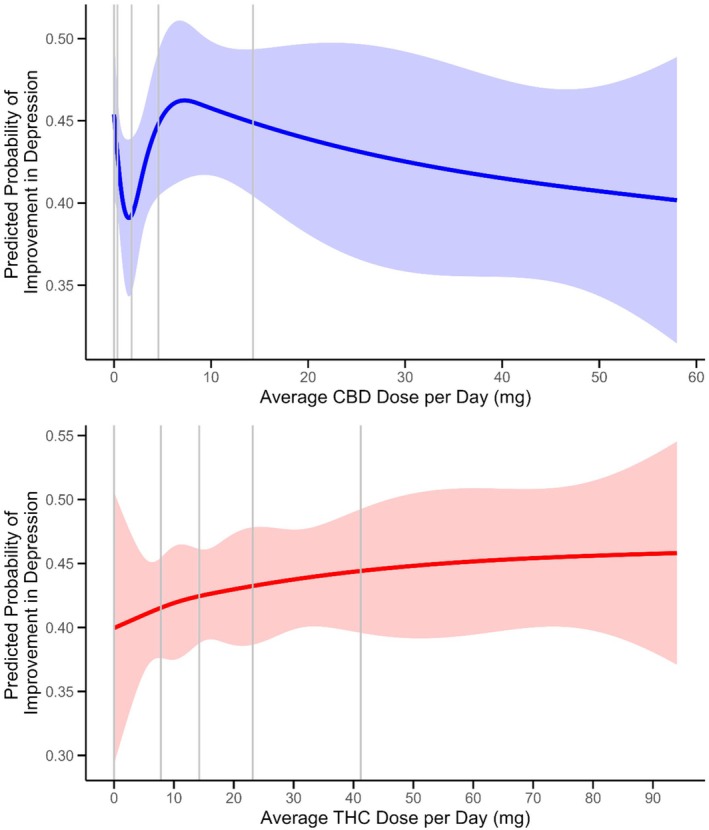
Predicted Probability of Achieving a 30% or Greater Improvement in Disturbed Sleep for Average Dose per Day (mg) for THC and CBD. Predicted probability using restricted cubic spline with knots at 5, 27.5, 50, 72.5, and 95 percentiles are shown with 95% confidence limits. Vertical gray lines mark quintile cutpoints for average dose.

The average percent improvement in depression symptom scores was 25.6%. The THC dose, CBD dose, and THC: CBD ratio were not significantly associated with a change in reported depressive symptoms among cancer patients (Appendix B, Figure A2). Appendix B, Figure A5 shows graphs of the predicted probability of achieving a 30% or greater improvement in depression for average dose per day (mg) for THC and CBD using restricted cubic spline with knots at 5, 27.5, 50, 72.5, and 95 percentiles. Appendix B, Figure A6 shows graphs of the predicted improvement in depression.

However, the use of any enteral products was associated with an improvement in depressive symptoms (1.36 reduction in depression score) compared to only the use of non‐enteral products (1.10 reduction, *p* = 0.04). Additionally, patients who reported using both enteral and topical products reported a greater improvement in depressive symptoms compared to those who used only enteral products (1.80 vs. 1.18 score improvement, Table 2).

## 4 Discussion

Among cancer patients in this study, we observed an improvement in symptoms of anxiety and depression for individuals who remained in the MMCP for more than 30 days and purchased enteral cannabis products. Overall, the THC dose was not strongly associated with anxiety or depression, while greater CBD doses were associated with greater improvements in anxiety but not depression scores.

This study suggests that the dose of CBD, the ratio of THC to CBD, and the route of administration of cannabis may all influence self‐reported anxiety in cancer survivors. Specifically, our study identified a positive association between CBD and reduced anxiety, consistent with existing literature [18]. The anxiolytic effects of cannabis products were more pronounced with enteral products, which had higher CBD levels than any other route of administration in this study. Previous studies with inverse U‐shaped dose–response curves have concluded that 300 mg may be the most efficacious CBD dose for anxiety [33]. Additional studies are needed to confirm whether providers may want to encourage cancer survivors to try cannabis products with relatively high CBD relative to THC doses and the use of enteral products for anxiety relief. Additionally, on the ESAS scales, a 1‐point improvement is considered clinically significant for anxiety [30]. So, while this study found a significant relationship between CBD and anxiety reduction, clinical significance may be modest.

Interestingly, THC dose was not significantly associated with a change in anxiety for the patients in our study. This contradicts clinical studies that have suggested that THC may have anxiogenic effects, especially at high doses [34]. Several factors may have contributed to these findings. For instance, cancer‐associated differences in the endocannabinoid system may have reduced THC‐related changes in anxiety [35]. Furthermore, prolonged use of THC may lead to the development of tolerance, potentially resulting in diminished effects on anxiety in regular users [36].

Our findings suggest that enteral products play an important role in improving feelings of depression. A key feature of enteral products is their ability to produce sustained effects over a longer period of time relative to other routes of administration; the duration of effects of enteral products is around 4–12 h [37]. The maximum effect of enteral products may be less predictable due to delayed onset [38]. Enteral products also can be more intense than other routes due to the conversion of THC to its more potent active metabolite, 11‐hydroxy‐tetrahydrocannabinol (11‐hydroxy‐THC), in the liver [39]. The use of topical products in addition to enteral products may have resulted in greater depressive symptom reduction due to the possible soothing effects associated with massaging a topical product into the skin [40, 41].

Existing literature supports the potential for CBD to relieve depressive symptoms, an effect seen in this study [42]. Conversely, THC is thought to reduce CB1 receptor activity over time, leading to diminished therapeutic impacts and potentially contributing to depression [43]. For this study, we only evaluated symptom scores after day 30 of MMCP enrollment. Any initial changes in depression related to THC may have dissipated before the 30‐day time point.

The implications of these findings for clinical practice are to move cannabinoid prescribing for cancer‐related mood symptoms toward more structured protocols. This study supports a CBD‐dominant, enteral‐first approach for patients with persistent psychological distress. Because there are no existing guidelines for cannabis use for anxiety in cancer patients, a helpful starting point for cannabinoid‐based anxiety relief may be the existing guidelines for cannabinoid titration in pain management, which recommend initiating CBD at low doses (e.g., 5 mg twice daily) and gradually titrating upward while monitoring tolerability and symptom response [44]. However, this must be tested in a clinical trial. While titrating, it may be helpful to implement the use of validated assessment scales, such as the ESAS used in this study, at baseline and at regular intervals. Safety monitoring should include assessment of unwanted cognitive effects and interactions with concurrent medications such as warfarin, buprenorphine, and tacrolimus [45]. Discontinuation should be considered if a clinically significant improvement is not achieved within the trial period.

A limitation of our dataset is that the cannabis transaction data were used as a proxy for product use and adherence, despite possible variability in use [46]. However, transaction data may be more accurate than patient‐reported dose estimates of cannabis, which are often inaccurate [47]. Objective transaction data from dispensaries allow for more precise tracking of the timing and contents of cannabis products for each patient. This, in turn, enhances confidence in inferences drawn from patient‐reported symptom responses, as standardized patient surveys were collected with each purchase. A second limitation is that patient cannabis use at baseline, prior use, and use outside of the MMCP purchases was unknown. Patients with prior cannabis use may have been able to tolerate greater doses and may have even required greater doses to perceive any effect compared to patients without previous exposure. Moreover, this dataset does not include a systematically collected objective assessment of what anxiety or depression medications were used by patients, nor adverse effects, which could have impacted symptom scores over time. Lastly, self‐reported depression and anxiety levels were subjective and not evaluated clinically [48].

Patients were recruited exclusively from Minnesota, which may reduce the generalizability of these findings to other patient populations. Even within a region, the differences between patients' cancer type, stage of cancer, and treatment regimens may affect neuropsychiatric symptom severity and sensitivity to CBD and THC. In this study, most patients did not report whether their cancer was metastatic, which may have impacted their symptom scores [49]. Also, the requirement of two post‐30‐day visits may have introduced selection bias for those who had greater longevity.

Future studies must account for the heterogeneity of cancer patients in general. Age, comorbidities, prior cannabis experience, and genetic variability in cannabinoid receptor expression in this population may impact the efficacy and tolerability of cannabinoids [50, 51, 52]. Even the preference for specific routes of administration may be confounded by patient characteristics; for example, fast‐acting inhalation or oromucosal products may be preferred by patients with more severe acute‐onset symptoms [53]. The integration of these factors into research and clinical decision‐making can help optimize therapeutic benefits, minimize risks, and advance more patient‐centered care in oncology.

## 5 Conclusions

This is the first large‐scale study in Minnesota to retrospectively examine cancer patients' reported anxiety and depression symptom responses to medical cannabis. We found that anxiety symptom responses varied according to the dose of cannabis, the ratio of THC to CBD, and the route of administration of cannabis products, and that depression symptom response was limited to patients using the enteral route of administration. Given the high prevalence of anxiety and depression symptoms among cancer patients, along with the potential for cannabis products to alleviate these serious psychological symptoms, this study suggests specific patterns of use that may improve the quality of life of cancer survivors.

## Author Contributions

All authors contributed to the study conception and design. Apoorva C. Reddy and Susan J. Park prepared the materials and collected the data. Data cleaning and analysis were performed by Apoorva C. Reddy, John M. Hampton, Susan J. Park, Faith Dickerson, Betty Chewning, Janvi Shah, Natalie Schmitz, and Amy Trentham‐Dietz. The first draft of the manuscript was written by Apoorva C. Reddy, and all authors commented on previous versions of the manuscript. All authors read and approved the final manuscript.

## Ethics Statement

This study was conducted in accordance with the ethical principles outlined in the Declaration of Helsinki and was approved by the University of Wisconsin Health Sciences Institutional Review Board (UW IRB). The UW IRB determined that this study is not research involving human subjects as defined by the Department of Health and Human Services and Federal Drug Administration regulations. The researchers obtained a waiver of consent due to minimal risks to the study participants including the use of limited coded data.

## Conflicts of Interest

The authors declare no conflicts of interest.

## Figures and Tables

**FIGURE A1:**
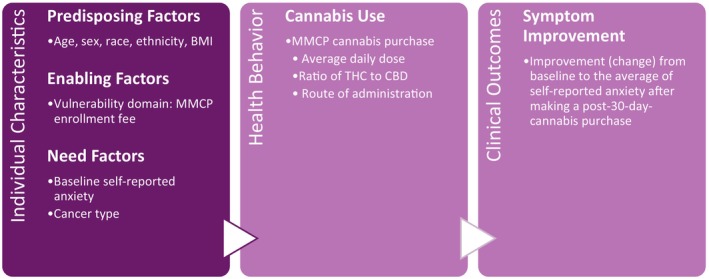
Gelberg–Andersen Model Applied to Cannabis Use and Self‐Reported Anxiety.

## Data Availability

The data that support the findings of this study are available on request from the corresponding author. The data are not publicly available due to privacy or ethical restrictions.

## Appendix A

### Applying the Gelberg

#### Andersen Behavioral Model

The Gelberg–Andersen model comprises four components: Predisposing factors, enabling resources, the need for care, and vulnerabilities [31]. Consistent with the Gelberg–Andersen Model, independent variables included demographics as characterized by the predisposing factors, the initial symptom score as characterized by the need for care domain, financial status (as characterized by the MMCP enrollment fee), which is an enabling resource and a vulnerability domain, the characteristics of the cannabis used (dose, ratio of THC to CBD, route of administration) as characterized by the cannabis use domain (Figure A1).

Vulnerability domains refer to characteristics of an individual or their environment that affect each of the three components defined above: Predisposition to, access to, and need for cannabis product use. Receiving public benefits (e.g., a reduced fee for cannabis products) can indicate vulnerability experienced by patients.

Recently, the model has been expanded and used successfully to examine mental health care access and outcomes in relation to the domains outlined above [31, 54]. The expanded Gelberg–Andersen Behavioral Model provides a framework for exploring the outcome of reported anxiety and depression symptom responses in relation to the use of cannabis products received by the patients enrolled in the Minnesota Medical Cannabis Program.

### Individual Characteristics

#### Predisposing Factors

##### Age

Age is a continuous variable defined as the age of patients at baseline as calculated by MMCP at the time of their enrollment.

##### Sex

Patient responses for sex include Male, Female, or Prefer not to answer.

##### Race and Hispanic ethnicity

Patient responses for race include Native American, Asian, Black, Unknown, Hawaiian/Pacific Islander, White, or Multiracial. Patients could indicate their Hispanic ethnicity with response options: Yes, No, Do not wish to answer, or Unknown.

##### BMI

Body mass index (BMI) is calculated by dividing a patient's baseline weight (pounds) by the square of a patient's height (inches).

#### Enabling Factors

##### MMCP enrollment fee

For reduced MMCP enrollment fee, the response options were N or Y. N indicates patient paid full MMCP enrollment fee ($200). Y indicates patient had a form of medical assistance that qualified them for the reduced MMCP enrollment fee ($50). This is also a vulnerability domain because payment of the reduced fee can be used as a proxy for their financial status.

#### Need Factors

Baseline self‐reported anxiety score

### Health Behavior

#### Cannabis Use

##### Average daily dose

To test average daily dose, if a patient purchases multiple products (regardless of route of administration) at once, their doses were divided by day supply and then summed to calculate a single average daily dose. The most common doses resulting in a 30% or greater symptom improvement were calculated and described descriptively. Average daily dose was included in multivariate regression as a continuous variable.

##### Ratio of THC to CBD

To test the ratio, if a patient purchased multiple products at once, the amount of THC was summed and the amount of CBD was summed to calculate the total amount of THC and total amount of CBD. The most common ratios resulting in a 30% or greater symptom improvement were calculated and described descriptively.

Route of administration: The most common routes of administration resulting in a 30% or greater symptom improvement were calculated and described descriptively. The route of administration was included as a categorical variable. To test routes of administration, dummy variables were created for each combination of the routes of administration. Combinations that patients less frequently used were bundled together into one category prior to running the regression.

### Outcome Variable

The primary outcome variable used in this study is the improvement (change) from baseline to the average of anxiety symptom scores reported after a product purchase has been made after 30 days of MMCP enrollment. By taking an average, patients who vary in their number of completions of the Patient Self‐Evaluation could be included. Thus, the 120‐day limit prevented the misattribution of changes in symptom score to cannabis products or use. Additionally, if a patient purchased raw cannabis, only their purchases prior to that purchase were eligible for inclusion in the analysis.

## Appendix B

### Additional Results

#### Cannabis Product Characteristics and Anxiety

The mean of the baseline anxiety score was 5.76 out of 10. The mean reduction (change) in anxiety was 1.43 from baseline to the average of scores post day‐30 cannabis purchase.

**TABLE A1:** Odds ratios and 95% confidence intervals for the Association between CBD quantity and Improvement of 30% or greater in Anxiety.

Average CBD Quantity Per Day (mg)	Improvement in Anxiety Score		Odds Ratio*	95% CI*	*p**
	< 30%	> = 30%			
Quintile 1: 0–0.36	221	171	0.84	0.63–1.12	0.23
Quintile 2: 0.37–1.81	242	151	0.68	0.51–0.92	0.01
Quintile 3: 1.82–4.58	244	148	0.66	0.49–0.88	0.05
Quintile 4: 4.58–14.32	217	176	0.89	0.67–1.18	0.42
Quintile 5: > 14.32	206	186	1 (ref.)		
*P*‐trend					0.06
Continuous (per mg)^a^			1.005	0.999–1.011	0.09

*Note:* (*) Adjusted for sex, age, race, ethnicity, BMI, and MMCP enrollment fee.

Abbreviations: CBD, cannabidiol; CI, confidence interval; MMCP, Minnesota Medical Cannabis Program; ref, reference; THC, tetrahydrocannabinol.

^a^ For continuous linear regression, values for THC and CBD were winsorized at the 95th percentile.

**TABLE A2:** Odds ratios and 95% confidence intervals for the Association between THC: CBD Ratio and Improvement of 30% or Greater in Self‐Reported Anxiety Scores.

THC:CBD ratio	Improvement in anxiety score		Odds ratio*	95% CI*	*p**
	< 30%	> = 30%			
Quintile 1: 0–1.11	211	181	1 (ref.)		
Quintile 2: 1.12–3.84	228	165	0.84	0.63–1.12	0.23
Quintile 3: 3.85–16.87	232	160	0.79	0.59–1.05	0.10
Quintile 4: 16.88–59.33	248	145	0.65	0.48–0.88	0.005
Quintile 5: > 59.33	211	181	0.94	0.71–1.26	0.69
*P*‐trend					0.32

*Note:* (*)Adjusted for sex, age, race, ethnicity, BMI, and MMCP enrollment fee.

Abbreviations: CBD: cannabidiol; CI confidence interval; MMCP: Minnesota Medical Cannabis Program; ref: reference; THC: tetrahydrocannabinol.

### Cannabis Product Characteristics and Depression

The mean of the baseline depression score was 4.72 out of 10. The mean reduction (change) was 1.21 from baseline to the average of depression scores post day‐30 cannabis purchase.
